# Prostate-Specific Membrane Antigen-Targeted Antibody–Drug Conjugates: A Promising Approach for Metastatic Castration-Resistant Prostate Cancer

**DOI:** 10.3390/cells14070513

**Published:** 2025-03-30

**Authors:** Chia-Hsien Shih, Tzuo-Yi Hsieh, Wen-Wei Sung

**Affiliations:** 1 School of Medicine, Chung Shan Medical University, Taichung 40201, Taiwan; s0901086@gm.csmu.edu.tw (C.-H.S.); joe.hsieh46@gmail.com (T.-Y.H.); 2 Department of Urology, Chung Shan Medical University Hospital, Taichung 40201, Taiwan; 3 Institute of Medicine, Chung Shan Medical University, Taichung 40201, Taiwan

**Keywords:** prostate neoplasm, metastasis, target therapy, chemotherapy, systemic therapy

## Abstract

Prostate cancer (PCa), especially metastatic castration-resistant prostate cancer (mCRPC), is a significant cancer characterized by its poor prognosis and limited treatment options. Prostate-specific membrane antigen (PSMA) has emerged as a diagnostic and therapeutic target for PCa due to its restricted expression in malignant prostate tissues. In this case, several PSMA-targeting molecules were developed for radiotherapy and immunotherapy. Antibody–drug conjugates (ADCs) are a novel therapeutic approach for various carcinomas that can selectively target PSMA-positive tumor cells and minimize off-target toxicity. ADCs have made great progress in the treatment of breast and bladder cancers, and some have received FDA approval for target therapy. However, studies on PSMA ADCs are limited, and most clinical trials are at stage I or II. Therefore, this study reviewed trials about PSMA-targeting ADCs for the treatment of PCa. Clinical trials have reported a favorable pharmacokinetic profile and antitumor activity. Toxicity studies have revealed manageable adverse effects, with no significant off-target toxicity in PSMA-negative tissues. This study highlights the therapeutic potential of PSMA ADCs for the treatment of mCRPC. However, it also emphasizes the necessity of further clinical investigation to optimize efficacy, safety, and patient outcomes.

## 1. Introduction

Prostate cancer (PCa) is the second most common cancer in males after lung cancer, with an estimated 1,600,000 cases and 366,000 deaths annually. This cancer accounts for 7% of newly diagnosed cancers in men worldwide and is one of the leading causes of cancer-related deaths among men in developed regions [1]. The prognosis for an individual with PCa is highly variable and dependent on the tumor grade and stage at primary diagnosis. Current methods for early detection, such as prostate-specific antigen (PSA) testing and digital rectal examination (DRE), allow for the diagnosis of most men when the disease is in its early stages. About 80% of men are diagnosed with organ-confined PCa, 5% with locoregional metastases, and 5% with distant metastases. For men with localized PCa, the potential for a long life is high, with a 10-year survival rate reaching as high as 99% if the cancer is detected early. However, men who are diagnosed with advanced-stage PCa, characterized by distant metastases, have a much bleaker prognosis, with only a 30% overall survival (OS) rate at 5 years [2].

Androgen deprivation therapy, often combined with an androgen receptor pathway inhibitor, represents a standard initial treatment approach for males diagnosed with advanced PCa. However, a significant majority of patients eventually experience disease progression even while undergoing hormonal therapies, leading to the clinical state known as metastatic castration-resistant PCa (mCRPC) [3]. The precise mechanisms underlying the transition from androgen-dependent (referred to as hormone-sensitive or castration-sensitive) PCa to nonmetastatic castration-resistant PCa (nmCRPC) remain largely elusive. mCRPC has a poor prognosis, with an expected median survival of only 2 to 3 years [4]. For males with metastatic and nonmetastatic nmCRPC, multiple active life-prolonging therapeutic options have emerged in the last decade. This article is intended to review novel radiotherapy and antibody–drug conjugates (ADCs) for the treatment of nmCRPC and mCRPC.

## 2. Prostate-Specific Membrane Antigens

Prostate-specific membrane antigen (PSMA) is a type II membrane protein that functions as a glutamate-preferring carboxypeptidase in all forms of prostate tissue [5]. PSMA expression is also found in the salivary and lacrimal glands, liver, spleen, bowel, kidneys, and sympathetic ganglia. Studies have found that PSMA is highly overexpressed in PCa around 100 to 1000 times the normal level, especially in advanced cancer. In Sven Perner’s study [6], the expression levels of PSMA exhibited statistically significant differences (*p* < 0.001) across benign prostatic tissue, localized PCa, and lymph node metastases. It was observed that elevated levels of PSMA and the presence of metastases correlated with the time of PSA recurrence (HR, 1.4; 95% confidence interval, 1.1–2.8; and *p* = 0.017 and hazard ratio, 5; 95% confidence interval, 2.6–9.7; and *p* < 0.001, respectively). This finding represents the diagnostic and therapeutic value of PSMA, which is used as a targeted antigen.

## 3. Antibody–Drug Conjugates (ADCs)

**Figure 1:**
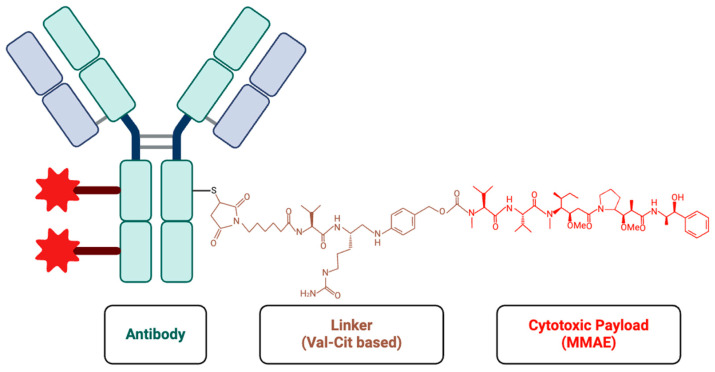
The structure of an ADC is composed of three key components: an antibody (green), a linker (brown), and a cytotoxic payload(red). The antibody is designed to specifically recognize and bind to tumor antigens. The linker is the bridge between the antibody and the cytotoxic pay-load. The cytotoxic payload (such as MMAE) is a potent agent that disrupts cellular processes, leading to cell death. This structure insures the selective delivery of the payload to tumor cells and minimizes the off-target effects.

ADCs have emerged as an innovative and promising approach in targeted cancer therapy. ADCs combine monoclonal antibodies, which specifically target tumor antigens, with potent cytotoxic agents to selectively destroy cancer cells while reducing off-target toxicity [7] (Figure 1). Initially, ADCs were primarily used to treat breast cancer and malignant lymphoma, such as gemtuzumab ozogamicin for acute myeloid leukemia and trastuzumab emtansine (T-DM1) for HER2-positive breast cancer [8]. More recently, ADCs such as enfortumab vedotin (approved in 2019) and sacituzumab govitecan (approved in 2021) have received FDA approval for UC treatment [9], highlighting the growing role of ADCs in the field of urology.

ADCs consist of three main components: an antibody, a linker, and a cytotoxic drug (payload). In ADC design, selecting stable, antigen-specific antibodies is critical for ensuring precise drug delivery to tumor cells while avoiding healthy tissues. These antibodies must distinguish between tumor and normal cells to prevent off-target toxicity. Humanized IgG is commonly used in ADCs to reduce immune reactions associated with non-human antibodies [10].

Linkers connect the antibody to the cytotoxic drug and play a crucial role in the release of drugs. Cleavable linkers are sensitive to the intracellular microenvironment, which releases the drug via hydrolysis or protease cleavage within endosomes. While non-cleavable linkers represent greater stability, on the other hand, they only release the cytotoxic drug into the lysosome [11].

**Figure 2:**
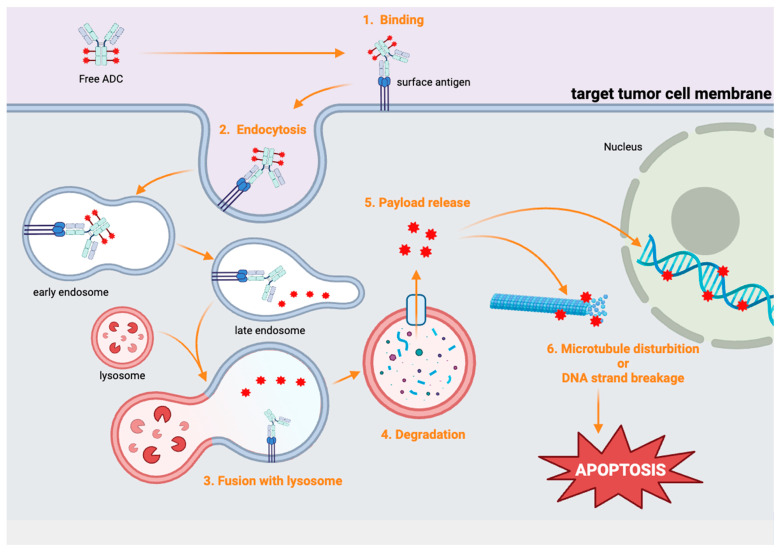
The mechanism of ADCs involves several sequential steps. First, the antibody component of the ADC specifically binds to the target antigen on the surface of the tumor cell membrane. After binding, the ADC is internalized into the cell through endocytosis, forming an early endo-some (blue membrane). This early endosome then becomes a late endosome, which eventually fuses with a lysosome (red membrane). Inside the lysosome, the ADC is degraded by lysosomal enzymes (red Pac-man), and the cytotoxic payload (red star polygon) is released. The free payload disrupts the formation of microtubules or leads to DNA strand breakage, which eventually induces apoptosis of the tumor cell.

The payload is a potent cytotoxic drug that is delivered directly into tumor cells in high concentrations. These payloads can be categorized as either microtubule disruptors or DNA-damaging agents. For instance, monomethyl auristatin E (MMAE) disrupts microtubule assembly and induces apoptosis [12]. SN-38 is a topoisomerase I inhibitor that causes DNA damage [13]. The use of these cytotoxic agents makes ADCs particularly effective at killing cancer cells (Figure 2).

## 4. Selective Cytotoxicity of ADCs

The mechanism of ADCs involves several critical steps that ensure selective cytotoxicity. First, the mAb component of the ADC specifically binds to the target antigen, such as PSMA on prostate cancer cells. This binding triggers receptor-mediated endocytosis, which leads to the internalization of the ADCs into tumor cells. Subsequently, ADCs are enclosed within an early endosome, which matures into a late endosome. The late endosome then fuses with a lysosome, where the ADC is exposed to lysosomal enzymes that cleave the linker. Upon cleavage, the free payload is released into the cytosol [14]. The payload then exerts its cytotoxic effect by either disrupting microtubule formation or inducing DNA strand breakage in the nucleus, ultimately leading to the apoptosis of tumor cells. This specific internalization and drug release process ensures that the cytotoxic agent is delivered to the tumor cells, minimizing the damage to healthy tissues.

However, the efficacy of ADCs can be reduced in tumors with heterogeneous antigen expression or poor internalization of the drug. Additionally, some ADC payloads exhibit a bystander effect. The released drug can affect nearby cells, potentially enhancing cytotoxicity in heterogeneous tumors but also increasing the risk of damaging normal tissues [15].

## 5. Anti-PSMA Monoclonal Antibodies

Compared with small molecule agents (~1.4 kD), such as Glu-urea-Lys in ^77^Lu-PSMA-617, monoclonal antibodies have a larger molecular weight (approximate 150 kDa). While a larger molecular weight could dramatically decrease the glomerular filtration rate of antibody-based drugs, this characteristic leads to a longer half-life [16]. Otherwise, several studies have shown that antibodies demonstrate better tumor uptake and more uniform distribution, primarily due to an optimal balance between their in vivo half-life and vascular/tissue penetration abilities [17,18].

## 6. 7E11

The 7E11.C5 antibody is a murine monoclonal antibody that recognizes the intracellular domain of PSMA and was generated by immunizing mice with human PCa LNCaP cells [19]. Subsequent immunohistochemical studies confirmed its strong specificity for epithelial cells in prostate tissue, with only limited expression observed in other tissues, such as the brain, salivary glands, and small intestine [20]. The 7E11.C5 antibody binds to a linear epitope composed of the first six amino acids at the N-terminal of PSMA, which is located on the cytoplasmic side of the plasma membrane. This intracellular localization restricts its ability to detect living cells, as the epitope becomes accessible primarily in apoptotic or necrotic cells. This unique property forms the basis of its application in the ProstaScint™ imaging agent for the detection of PCa, although the requirement for substantial quantities of nonviable cells limits its diagnostic sensitivity, particularly for smaller tumors. Despite these limitations, 7E11.C5 has played a critical role in PSMA research, laying the foundation for the development of more advanced antibodies and therapeutic strategies targeting PSMA.

## 7. J591

J591 is the first humanized monoclonal antibody targeting the extracellular domain of PSMA, having shown potential in both diagnostic and therapeutic applications. When conjugated with the radionuclide lutetium-177, J591 delivers targeted radiotherapy by binding to PSMA, leading to internalization and radiation-induced cell death. Clinical trials with ^177^Lu-J591 have demonstrated its ability to reduce PSA levels and stabilize disease in a significant number of patients. Imaging studies confirmed tumor targeting, and PSA reductions of over 30% were seen in many patients [21].

225Ac-J591 is composed of the radioactive particles Actinium-225 and J591. A phase I trial recruited 32 patients with mCRPC and treated them with a single dose of 225Ac-J591. As a result, 46.9% of patients experienced a 50% decline in PSA, and 59.1% had a positive response in their circulating tumor cell (CTC) counts [22].

89Zr-DFO-J591 is a radiotracer for immunoPET imaging. In a study, 89Zr-labeled J591 demonstrated high radiochemical yield (>77%) and purity (>99%) and maintained strong immunoreactivity for up to 7 days. In vivo studies on mice with PSMA-positive tumor cells (LNCaP) showed significant tumor-specific uptake, reaching 45.8% after injection for 144 h. ImmunoPET imaging provided excellent tumor-to-muscle contrast, allowing the clear delineation of PSMA-positive tumors. The complex showed high in vivo stability and thermodynamic favorability, ensuring its reliable performance [23]. Overall, J591 shows great potential for both non-invasive imaging and radiotherapy in the treatment of PCa. Due to its high specificity for PCa cells and tumor uptake rate, J591 is a promising tool for enhancing the accuracy of diagnostic imaging and improving the efficacy of targeted radiotherapy in clinical applications.

## 8. 5D3

5D3 is a monoclonal antibody that specifically targets the extracellular domain of PSMA. Unlike earlier antibodies, such as J591, 5D3 binds specifically to surface-exposed conformational epitopes of native PSMA. It demonstrates high specificity, minimal cross-reactivity with unrelated proteins, and an approximately 10-fold higher affinity than J591 [24]. Moreover, the 5D3 Fab fragment achieved an even faster tumor-specific contrast in the NIRF image, which is visible within 2 h and sustained for 24 h. 5D3 is also implanted in 111In-DOTA-5D3 as a surrogate for therapeutic isotopes such as ^177^Lu or 90Y. In mice bearing PSMA (+) PC3 PIP xenografts, tumor uptake peaked at 24 h postinjection and remained high until 72 h. Compared with control experiments xenografted with the PSMA (−) PC3 flu cells, it revealed no specific binding up to 24 h after incubation [25]. These results highlight the tumor specificity of 5D3.

## 9. ADCs Targeting PSMA in PCa

The clinical trials of PSMA ADCs are summarized in Table 1.

### 9.1. PSMA-MMAE

#### 9.1.1. NCT01414283

NCT01414283 is an open-label, dose-escalation phase 1 clinical trial investigating PSMA ADCs in patients with mCRPC. The PSMA ADC consisted of a fully human anti-PSMA monoclonal antibody (mAb) conjugated to MMAE via a valine–citrulline linker, which was designed to be stable in blood but cleaved intracellularly in PSMA-expressing PCa cells. The mechanism of MMAE disrupts microtubule polymerization, inducing cell cycle arrest and cell death. The trial enrolled 52 patients between October 2008 and October 2012, all of whom had progressive mCRPC with prior taxane-based chemotherapy. Exclusion criteria included significant cardiac or pulmonary disease, active infections, prior PSMA-targeting therapies, or a history of substance abuse. PSMA-MMAE was administered intravenously every three weeks for up to four cycles. If the patients demonstrate clinical benefits, those eligible entered an extension study for up to 13 additional cycles (NCT01414296). The study was completed in September 2013.

The results of this trial were published by Daniel P Petrylak in Prostate [26]. The median age of the 52 patients was 70 years, and 62% of the patients had an ECOG performance status of 1. The study established the maximum tolerated dose (MTD) of PSMA-MMAE at 2.5 mg/kg, and dose-limiting toxicities (DLTs) were observed at 2.8 mg/kg. Common adverse effects included fatigue (40%), neutropenia (33%), nausea (29%), and temporary elevations in liver enzymes (25%). Pharmacokinetic analysis showed that peak serum concentrations of free MMAE were reached 2–4 days post-infusion, indicating the slow release from the ADC, and its half-life ranged from 2 to 3 days. Repeat dosing did not lead to a significant accumulation of the drug. PSMA-MMAE also demonstrated encouraging antitumor activity, particularly at doses ≥ 1.8 mg/kg. Among the treated cohort, eight patients achieved PSA reductions of ≥50%, with maximum declines exceeding 90% in two cases. Changes in CTC counts further demonstrated the treatment’s efficacy: eight patients experienced a shift from unfavorable (≥5 cells/7.5 mL of blood) to favorable (<5 cells/7.5 mL) CTC counts at doses ≥ 1.6 mg/kg.

Over half of the participants completed at least four treatment cycles, and 10 patients continued therapy in an extension phase for up to 11 months. These findings highlight the potential of PSMA ADCs as a therapeutic option for mCRPC, with a favorable safety profile and evidence of significant antitumor activity at the recommended phase 2 dose of 2.5 mg/kg.

#### 9.1.2. NCT01695044

NCT01695044 is an open-label, single-arm, multicenter phase 2 clinical trial evaluating the efficacy and tolerability of PSMA-MMAE in patients with mCRPC, which is also a further phase 2 trial of NCT01414283. The trial enrolled 119 patients at 28 U.S. centers between September 2012 and October 2014, including chemotherapy-experienced and chemotherapy-naïve subjects who were required to have received and progressed on abiraterone acetate and/or enzalutamide. The enrolled patients are divided into two groups: (1) prior history of treatment with at least one taxane-containing chemotherapy regimen and (2) no prior history of treatment with a cytotoxic chemotherapy regimen but had received Radium-223. The patients received PSMA-MMAE at an initial dose of 2.5 mg/kg via intravenous infusion every three weeks for up to eight cycles, which was later adjusted to 2.3 mg/kg due to safety concerns, such as febrile neutropenia and sepsis. Subjects with clinical benefits after eight cycles could enter an extension study (NCT02020135). The study was completed in February 2015.

The results of this trial were published by Daniel P Petrylak in *Prostate* [27]. The median age of 119 patients was 71 years, and 96% of the participants had an ECOG performance status of 0 or 1. Efficacy analysis revealed PSA declines of ≥50% in 14% of all treated and 21% of chemotherapy-naïve subjects. CTC reductions of ≥50% were observed in 78% of patients (≥5 cells/7.5 mL of blood), and 47% achieved favorable CTC conversions (<5 cells/7.5 mL). Chemotherapy-naïve patients showed higher CTC response rates, with 89% demonstrating ≥50% declines and 53% achieving conversions compared with 74% and 45% in the chemotherapy-experienced group, respectively. Safety analysis indicated that 95% of patients experienced treatment-related adverse events (TRAEs), with 58% reporting grade 3 or higher events. The most common AEs included neutropenia (36%), fatigue (34%), decreased electrolytes (29%), anemia (25%), and peripheral neuropathy (12%). Peripheral neuropathy was the leading cause of treatment discontinuation, which was reported by 14 patients. Serious adverse events (SAEs) occurred in 51% of patients, and most of them suffered from dehydration, hyponatremia, or febrile neutropenia. Additionally, a smaller proportion of chemotherapy-naïve patients discontinued treatment before completing five cycles due to progressive disease or AEs (54% vs. 66%).

These findings demonstrate that PSMA-MMAE exhibits notable antitumor activity, with manageable toxicities at the recommended phase 2 dose of 2.3 mg/kg. Notably, chemotherapy-naïve patients showed more favorable outcomes based on greater PSA responses, CTC reductions, and OS (97.1% vs. 91.7%). The optimization of dose regimens and enhanced patient selection could further improve outcomes and minimize adverse effects.

### 9.2. ARX517

#### NCT04662580

NCT04662580 is a phase 1, multicenter, open-label trial investigating the safety, pharmacokinetics, pharmacodynamics, and preliminary antitumor activity of ARX517 in patients with mCRPC who are resistant or refractory to standard therapies. ARX517 consists of a humanized anti-PSMA monoclonal antibody (IgG1κ) linked to two proprietary microtubule-disrupting toxins (Amberstatin-269 and AS269). It utilizes a non-cleavable polyethylene glycol linker and stable oxime conjugation to minimize premature payload release. The study includes dose-escalation (phase 1a) and dose-expansion (phase 1b) stages to determine the MTD and RDDs. Eligible participants were men aged 18 or older with histologically confirmed prostate adenocarcinoma, metastatic disease, and castration-resistant PCa (serum testosterone ≤ 50 ng/dL). The patients were required to have received at least two prior lines of therapy, including one second-generation androgen receptor (AR) inhibitor (such as abiraterone, darolutamide, apalutamide, or enzalutamide). ARX517 is administered intravenously every three or four weeks.

Although this study is still recruiting participants, a current preclinical study has been published [28]. For in vitro testing, ARX517 demonstrated a selective cytotoxicity in PSMA-expressing PCa cell lines. In a 4-day proliferation assay, ARX517 exhibited subnanomolar activity (IC_50_ ≤ 0.5 nmol/L) in high-PSMA-expressing cell lines; on the contrary, in PSMA-low or PSMA-negative cell lines, the cytotoxic activity was significantly reduced (PC-3: IC_50_ > 30 mmol/L).

Among in vivo mouse xenograft models, male nu/nu mice were implanted with MDA-PCa-2b tumors. A single dose of ARX517 (5 and 10 mg/kg) resulted in up to 83% tumor growth inhibition (TGI), while an unconjugated mAb (10 mg/kg) and isotype control ADC (with AS269) showed no significant tumor suppression.

In enzalutamide-sensitive PCa PDX mice, the combination of ARX517 (3 mg/kg) and enzalutamide (10 mg/kg) showed an additive effect, with 85% TGI (*p* < 0.0001). In comparison, a single dose of ARX517 (3 mg/kg) or enzalutamide (10 mg/kg) only represented 66% and 41% TGI, respectively. In the enzalutamide-resistant C4-2 mice, a single dose of ARX517 (1 mg/kg) achieved 37% (*p* < 0.01) TGI, and a 5 mg/kg dose of ARX517 was able to lead to complete tumor suppression. Importantly, enzalutamide had no significant effect in this model, while ARX517 remained effective.

Preclinical studies demonstrated that ARX517 has a favorable tolerability profile with a wide therapeutic index. In Sprague–Dawley rats, single doses below 60 mg/kg were well tolerated, with only transient liver enzyme elevations and mild hematologic changes. In cynomolgus monkeys with repeat dosing up to 6 mg/kg (highest non-severely toxic dose), mild and reversible effects on liver enzymes, the spleen, or thymus was observed, and no significant cardiovascular, respiratory, or neurologic toxicity was observed. Importantly, plasma free payload levels remained >100-fold below cytotoxic IC_50_, which indicated the minimal off-target effects. Otherwise, ARX517 demonstrated a half-life of 13.5 days in cynomolgus monkeys, which is significantly longer than that of MLN-2704 (2.7 days) and MEDI3726 (0.3–1.8 days).

Recently, preliminary results of its phase I/II clinical trial have released [29]. The trial employed a dose-escalation design, with 24 patients who had previously received at least two FDA-approved therapies for mCRPC. These patients were administered with ARX517 every three weeks at escalating doses (0.32–2.88 mg/kg). The results indicated that the drug was generally well tolerated, with grade 1/2 TRAEs such as dry mouth (41.7%), fatigue (33.3%), and diarrhea (20.8%) being reported. Notably, there were no DLTs or serious AEs, and the occurrence of higher-grade adverse events was minimal. Importantly, in a higher-dose cohort, it revealed that >50% PSA (*n* = 8) and circulating tumor DNA ctDNA (*n* = 12) decline were observed. Pharmacokinetic profiles of both the total antibody and ADC suggested strong stability, indicating minimal premature payload release in circulation.

ARX517 demonstrated favorable tolerability, with manageable and reversible toxicities. Its superior systemic stability and prolonged half-life contribute to its wide therapeutic index. Moreover, the results of in vivo tests may suggest that the combination of enzalutamide (or other AR inhibitor) with ARX517 could provide a promising benefit in both patients sensitive and resistant to androgen deprivation therapy. Remarkably, the early clinical results demonstrate the potential of ARX517 as an effective and safe treatment option for pretreated mCRPC patients, warranting further investigation in subsequent clinical trials. Other human clinical trials are in progress to deeply assess its safety, pharmacokinetics, and therapeutic potential in mCRPC.

### 9.3. MLN2704

The structure of MLN2704 consists of a humanized mAb MLN591 targeting the external domain of PSMA, an antimicrotubule agent maytansinoid-1 (DM1), and linkage via a disulfide bond [30]. The phase 1/2 clinical trial of MLN2704 evaluated its safety, pharmacokinetics, and antitumor activity in patients with progressive mCRPC [31]. Sixty-two patients were enrolled and divided into four dosing schedules: weekly (60–165 mg/m^2^), every 2 weeks (120–330 mg/m^2^), every 3 weeks (330–426 mg/m^2^), and a 6-week cycle (330 mg/m^2^).

MLN2704 demonstrated limited clinical efficacy. Only 8% of patients represented ≥50% reduction in PSA levels, and most of them were in the dose of 330 mg/m^2^. No significant tumor regression was observed, although 35% of the patients exhibited stable disease. In addition, the major limiting factor for the utility of MLN2704 was its safety profile. Peripheral neuropathy was the most common adverse event (71%), with 10% of them experiencing severe (grade 3 or 4) neuropathy. Neurotoxicity is considered to be the instability of the disulfide linker between MLN591 and DM1. Pharmacokinetic analysis revealed rapid clearance of the conjugated antibody, while free DM1 levels persisted in circulation for up to 30 days. The levels of free DM1 were >20-fold higher in the 2-week and 3-week groups with the dose of 330 mg/m^2^. This instability resulted in the premature deconjugation of DM1 in the bloodstream, leading to elevated systemic exposure to free DM1 and subsequent toxicity.

The clinical development of MLN2704 was hampered by significant neurotoxicity and limited antitumor activity. This trial indicates that linker instability substantially narrowed the therapeutic window of ADCs. Advancements in linker technology could help reduce systemic toxicity and improve the efficacy of PSMA-targeting ADCs.

### 9.4. MEDI3726

#### NCT02991911

NCT02991911 is an open-label, dose-escalation phase 1/1b clinical trial evaluating the safety, pharmacokinetics, immunogenicity, and preliminary efficacy of MEDI3726 in patients with mCRPC. MEDI3726 is composed of a humanized mAb J591 conjugated to pyrrolobenzodiazepine (PBD) dimers via a linker [32]. PBD dimers are potent cytotoxins that form DNA interstrand crosslinks, which are minimally disrupted by DNA repair mechanisms [33].

The study enrolled 33 patients between February 2017 and November 2019, all with histologically confirmed mCRPC that had progressed after abiraterone, enzalutamide, and taxane-based chemotherapy. MEDI3726 was administered intravenously every three weeks at doses ranging from 0.015 to 0.3 mg/kg.

The result of this trial was published by Johann S. de Bono in *Clin Cancer Res* [34]. The median age of the 33 participants was 71 years. The MTD of MEDI3726 was not identified; however, the MAD was established at 0.3 mg/kg. Among the 33 patients, 90.9% experienced drug-related AEs, with common adverse events that included skin toxicities, effusions, and elevated liver enzymes. Grade 3/4 TRAEs were observed in 45.5% of patients, and 33.3% of patients discontinued treatment due to toxicity. Pharmacokinetics revealed nonlinear clearance and a short half-life of 0.3–1.8 days, while 40.6% of the patients developed antidrug antibodies. In an efficacy test, a 12.1% composite response rate was reported, with responses occurring in higher-dose cohorts (≥0.2 mg/kg). One patient (3%) exhibited a PSA reduction of ≥50%, and four patients (12%) achieved a confirmed CTC response in the low CTC group (≤50 CTC/7.5 mL blood). The median PFS was 3.6 months, and the OS was 8.9 months. This trial demonstrated a relatively limited activity of MEDI3726.

## 10. Optimizing PSMA-Targeted ADCs: Balancing Efficacy and Safety

ADCs have emerged as a promising therapeutic strategy for PCa, which has aroused interest in the development of PSMA-targeting therapy. PSMA is a critical diagnostic and therapeutic target due to its selective expression in PCa cells, particularly in the advanced and metastatic stages. Its overexpression provides a unique opportunity for the precise delivery of cytotoxic agents, maximizing antitumor efficacy while minimizing off-target toxicity. Several researchers have invested in the development and clinical trials of PSMA-targeting ADCs for mCRPC. Despite notable progress, challenges related to efficacy and safety remain significant barriers to their clinical adoption.

PSMA-targeting ADCs have demonstrated their ability to induce significant tumor responses. PSMA-MMAE showed promising antitumor activity in chemotherapy-naïve patients, with ≥50% PSA reductions observed in up to 21% cases and 53% of CTC conversion. This highlights its potential efficacy when administered at optimized doses (2.3 mg/kg). According to Johann S. de Bono’s article, the CTC is the independent predictor for the prognosis of mCRPC, with the OS improved in conversion to a favorable CTC [35]. In this case, the OS of chemotherapy-naïve patients was observed to be 97.1% over 7 months. These results underscore the antitumor activity and ability of PSMA ADCs to improve survival.

The use of advanced payloads, such as microtubule disruptors (MMAE) and PBD dimers, further enhances the efficacy of ADCs. These payloads are designed to disrupt cell division or induce DNA damage selectively in tumor cells, and most of them could bypass the resistance mechanism associated with conventional therapies. Despite these strengths, safety concerns have been a major limitation for PSMA-targeting ADCs. PSMA-MMAE showed DLTs, including febrile neutropenia and sepsis, which necessitated dose adjustments from 2.5 mg/kg to 2.3 mg/kg. MLN2704 was associated with high rates of peripheral neuropathy (71%). Similarly, MEDI3726 faced significant toxicity challenges, with 45.5% of patients experiencing grade 3/4 TRAEs. Pharmacokinetics plays a critical role in narrowing the therapeutic window of these agents. Rapid deconjugation of MLN2704 (associated with linker instability) and the short half-life of MEDI3726 (0.3–1.8 days) increase premature payload release and systemic exposure to free cytotoxic agents, resulting in the exacerbation of adverse effects.

To improve the therapeutic potential of ADCs, several strategies should be explored. The therapeutic efficacy of ADCs is correlated with the concentration and retention time of the payload within tumor cells, and exceeding a threshold concentration is essential for tumor stasis [36]. This requires a balance between ADC stability in circulation and efficient payload release in the tumor microenvironment. Parameters such as the conjugation site, linker length, cleavage mechanism, and steric hindrance play a crucial role in optimizing linker design [37]. A well-designed linker ensures effective payload delivery, enhancing therapeutic outcomes while minimizing systemic toxicity. Moreover, the development of payloads with lower systemic toxicity and the adjustment of dose regimens could improve patient tolerability. Modifying the physicochemical properties of the payload, particularly its polarity, can improve antibody aggregation, plasma stability, and bystander effects in ADCs [38]. Hydrophobic payloads enhance bystander effects by readily penetrating cell membranes, while the slow diffusion of hydrophilic payloads could potentially reduce the off-target toxicity [39]. Otherwise, selecting payloads with a small molecular weight, great tissue penetration, and short half-life is also a crucial point in ADC design.

## 11. Comparison Between PSMA-Targeting ADCs and ^177^Lu-PSMA Radiotherapy

Both PSMA-targeting ADCs and ^177^Lu-PSMA radiotherapy represent pioneering strategies in the treatment of mCRPC. These two approaches leverage the selective binding of PSMA to deliver targeted therapeutic agents directly to cancer cells. Despite this shared targeting mechanism, their underlying treatment modalities, efficacy profiles, and clinical applications diverge significantly (Table 2).

^177^Lu-PSMA-617 (Pluvicto^TM^) is a radiopharmaceutical product approved by the FDA for the treatment of PCa. The structure of ^177^Lu-PSMA-617 contains three components: (1) a PSMA-binding motif (Glu-urea-Lys), (2) a chelator (DOTA), and (3) a linker (comprising 2-naphthyl-L-alanine (Nal) and tranexamic acid (TXA)) [43]. This structure allows for DOTA to be chelated with lutetium-177, a beta-emitting radionuclide with a half-life of 6.64 days. Once injected into the body, the binding motif binds selectively to PSMA-positive cancer cells. The radioligand is then internalized into the cancer cells, where the beta particles emitted by lutetium-177 cause DNA damage and then lead to cell death [44]. On the contrary, ADCs selectively deliver toxic agents into PSMA-expressing cells, which enhanced the specificity and reduced systemic toxicity compared to traditional chemotherapy. However, the therapeutic efficacy of ADCs is relied on the efficient internalization of the conjugate, as well as sufficient and uniform PSMA expression across tumor sites. In contrast, ^177^Lu-PSMA-617 binds and internalizes to PSMA-positive cells selectively, where the emitted beta particles induce cell death. This radiotherapy modality offers an advantage with its crossfire effect; on the other hand, radiation can affect neighboring cancer cells even if they express lower levels of PSMA [45]. Nevertheless, one challenge associated with radiotherapy is the potential toxicity in organs with PSMA expression, such as the kidneys and salivary glands [46].

Clinically, ^177^Lu-PSMA-617 has been shown to improve progression-free survival (PFS) and overall survival (OS) in mCRPC patients, particularly in those who have previously received androgen receptor-directed therapies and taxane chemotherapy [47]. The phase III VISION trial validated the benefits of ^177^Lu-PSMA-617 by showing significant improvements in the median radiographic PFS (8.7 vs. 3.4 months) and median OS (15.3 vs. 11.3 months) compared with standard care [42]. By comparison, PSMA-targeting ADCs such as ARX517 show promising results in later-line mCRPC treatment, with early-phase trials indicating a PSA response rate of 40–50% in higher-dose cohorts. The treatment is generally well tolerated, with mild adverse effects and no dose-limiting toxicities reported so far. Ongoing trials are investigating its potential in combination with other therapies, and further phase III studies are needed to confirm its efficacy and long-term safety in mCRPC.

The full clinical benefits of PSMA-targeting ADCs remain uncertain, and further phase III trials are needed to determine their role in the treatment of mCRPC. Unlike ^177^Lu-PSMA-617, whose efficacy and safety are already validated in large-scale trials, PSMA-ADCs are still in the early stages of clinical exploration, emphasizing the necessity to assess their long-term efficacy and safety in diverse dose regimes and patient populations. The clinical outcomes of PSMA ADCs, including PSA response rates and toxicity profiles, are summarized in Table 3.

## 12. Future Perspectives: Optimizing PSMA-Targeting ADCs for mCRPC

**Figure 3:**
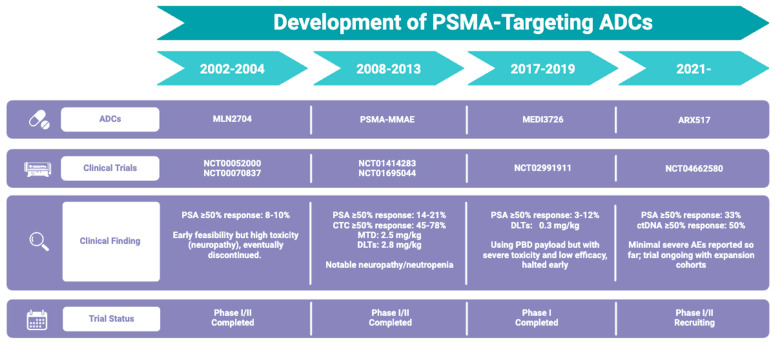
Summary of development of PSMA-targeting ADCs.

ADCs targeting PSMA have emerged as a promising therapeutic approach for metastatic castration-resistant prostate cancer (mCRPC). By leveraging the tumor-specific overexpression of PSMA, ADCs offer a means to deliver potent cytotoxic agents directly to PCa cells while minimizing off-target toxicity. Several clinical trials have demonstrated encouraging antitumor activity, particularly in chemotherapy-naïve patients, with significant reductions in PSA levels and CTC counts (Figure 3).

However, the clinical development of PSMA-targeting ADCs has faced challenges. Clinical trials have reported suboptimal outcomes due to ADC instability, ADC-associated AEs, and limited therapeutic efficacy. One major limitation is the instability of ADCs, which leads to premature drug release and systemic toxicity. For instance, MLN2704 suffered from disulfide linker instability, resulting in the rapid deconjugation of DM1 in circulation. It led to high systemic exposure and severe peripheral neuropathy, which ultimately halted its clinical development. Another challenge is ADC-associated AEs, particularly hematologic toxicities and peripheral neuropathy, which result in DLTs. For example, PSMA-MMAE (NCT01695044) showed promising antitumor activity, but adjustment of the dose regime is unavoidable due to high rates of neutropenia (36%), anemia (25%), and peripheral neuropathy (12%). MEDI3726 exhibited severe skin toxicity and hepatotoxicity as well, leading to early termination of the trial. Notably, limited therapeutic efficacy is also a barrier for treatment of mCRPC. While PSMA-MMAE demonstrated PSA reductions in some patients (≥50% in 14–21%), the overall response rates remained modest. A major factor is heterogeneous PSMA expression, where tumors with low or heterogeneous PSMA levels may not effectively internalize the ADC. Moreover, payload resistance, such as the upregulation of drug efflux pumps like P-glycoprotein or DNA repair mechanisms, could reduce the cytotoxicity of ADCs.

The future of PSMA-targeting ADCs focuses on improving efficacy, reducing toxicities, and enhancing targeting mechanisms.

Preclinical studies suggest that combining ADCs with AR inhibitors, PARP inhibitors, or immune checkpoint inhibitors may enhance tumor cell inhibition. AR inhibitors can sensitize tumor cells to ADC cytotoxicity, while PARP inhibitors exploit DNA repair deficiencies, making tumors more vulnerable [48]. ADCs combined with immune checkpoint inhibitors may also boost the immune response. Additionally, ADCs that induce immunogenic cell death (ICD) can make tumors more responsive to subsequent immune therapies, creating a synergistic effect that improves overall outcomes [49].

Dose-limiting toxicities, particularly hematologic and neurologic side effects, remain a challenge for ADC therapies. Strategies like intervention with prophylactic G-CSF to prevent neutropenia and the early detection of neuropathy can help manage these risks [50]. Flexible dose adjustments based on individual tolerance are crucial for maintaining efficacy while minimizing AEs. These approaches will make ADC therapies safer and more applicable for long-term use, especially when combined with other treatments.

Next-generation ADCs are dedicated to site-specific conjugation and controlled release mechanisms. Site-specific conjugation ensures more stable and precise attachment of the payload to the antibody, reducing off-target release. Additionally, protease-sensitive linkers enable the payload to activate only within the tumor microenvironment, which could also reduce systemic exposure. Otherwise, payload selection also plays an important role in ADC efficacy. Although the physicochemical properties of ADCs limit the choice in payloads, favorable solubility, amenability to conjugation, and stability are basic criteria for ADC design [51].

Although PSMA is widely recognized as a promising target for prostate cancer therapy, increasing evidence suggests that PSMA expression is moderately heterogeneous. A study analyzing 51 patients with both primary PCa and distant metastases found significant variability in PSMA expression [52]. As result, 7 out of 51 (13.7%) primary tumors and 6 out of 51 (11.8%) metastases displayed heterogeneous staining patterns. More importantly, two primary tumors and eight metastases (total: 10/51, 19.6%) exhibited PSMA-negative staining (<10% positive tumor cells), indicating that nearly 20% of patients may not be suitable candidates for PSMA-targeted therapies. This heterogeneity was observed across different tumor differentiation patterns, and no clear correlation was found between PSMA expression levels and histological parameters such as the Gleason score or metastatic site. Additionally, microenvironmental factors within the tumor may modulate PSMA expression, contributing to dynamic changes that further complicate targeted treatment approaches.

These findings highlight the critical need for PSMA screening before initiating PSMA-targeted therapies to identify PSMA-low or PSMA-negative patients who may not respond effectively. Notably, several studies found that androgen deprivation therapy can result in increased PSMA expression in patients with low PSMA levels [53]. Future strategies could involve combination therapies with androgen receptor inhibitors to enhance PSMA expression and improve treatment efficacy.

Despite the challenges, PSMA-targeting ADCs represent a significant step forward in the treatment landscape of mCRPC. Future research should focus on refining ADC design, optimizing dosing regimens, and integrating these agents with existing PCa therapies such as androgen receptor inhibitors and radioligand therapies. With continued innovation and clinical validation, PSMA ADCs have the potential to become a key component of precision medicine for patients with advanced PCa, offering improved survival and quality of life.

## 13. Conclusions

In conclusion, PSMA-targeted ADCs represent a promising advancement in the treatment of metastatic castration-resistant prostate cancer (mCRPC). By exploiting the selective overexpression of PSMA on prostate cancer cells, these agents offer a targeted approach that can deliver potent cytotoxic drugs directly to tumors while reducing off-target toxicity. Clinical studies of various PSMA ADCs have demonstrated encouraging antitumor activity and manageable safety profiles, particularly in chemotherapy-naïve patients. However, challenges such as ADC instability, heterogeneous PSMA expression, and dose-limiting toxicities continue to limit their clinical application. Future research should focus on refining ADC design—through improved linker stability, optimized payload selection, and site-specific conjugation—and on exploring combination strategies with established therapies like androgen receptor inhibitors and immune checkpoint inhibitors. With continued innovation and thorough clinical validation, PSMA-targeted ADCs have the potential to become an integral component of precision medicine for patients with advanced prostate cancer, ultimately improving their survival and quality of life.

## Figures and Tables

**Table 1:** Clinical trials of PSMA ADCs.

ADC Name	Antibody	Linker Type	Drug Payload	Clinical Trial	Stage	Years	Inclusion Criteria	Enrollment	Status
PSMA-MMAE	Fully human anti-PSMA mAb	Valine–citrulline linker	MMAE	NCT01414283	Phase 1	2008–2013	Patients with mCRPC who have received prior chemotherapy and/or androgen receptor inhibitors; measurable disease required	52	Completed
				NCT01695044	Phase 2	2012–2015		119	Completed
ARX517	Humanized anti-PSMA IgG1κ	Non-cleavable polyethylene glycol linker	AS269	NCT04662580	Phase 1/2	2021 till now	Patients with mCRPC who have received ≥ 2 prior lines of therapy, including AR inhibitors and chemotherapy	352 (estimated)	Recruiting
MLN2704	Humanized MLN591	Disulfide linker	Maytansinoid-1	NCT00052000	Phase 1	2002–2004	Patients with mCRPC with failed hormonal therapy	29	Completed
				NCT00070837	Phase 1/2	2003–2004		46	Completed
MEDI3726	Humanized J591	Cleavable linker	PBD dimer	NCT02991911	Phase 1	2017–2019	Patients with mCRPC after prior receipt of abiraterone and/or enzalutamide and taxane-based chemotherapy	33	Completed

Abbreviation: PSMA, prostate-specific membrane antigen; ADC, antibody–drug conjugates; MMAE, monomethyl auristatin E; mCRPC, metastatic castration-resistant prostate cancer; AR, androgen receptor; PBD, pyrrolobenzodiazepine.

**Table 2:** Comparison of prostate cancer treatment modalities.

Therapy	Mechanism	Reported Clinical Efficacy	Common Toxicities	Clinical Role
AR inhibitors (e.g., abiraterone and enzalutamide)	Inhibit androgen production or AR pathway signaling	~4–5 mo OS benefit; high PSA response in early mCRPC [40]	Hypertension, fatigue, and liver enzyme elevations	First line in advanced PC; most of patients develop AR resistance
Chemotherapy (docetaxel and cabazitaxel)	Systemic cytotoxicity via microtubule disruption	~2–3 mo OS benefit vs. control; ~30–45% PSA decline [41]	Neutropenia, neuropathy, and alopecia	Standard second line after AR inhibitors; broad cytotoxic approach
^177^Lu-PSMA radioligand therapy	Beta-emitting isotope targeting PSMA-positive tumor cells	OS prolongation (phase III VISION trial: ~4 mo); 50–80% PSA decline [42]	Fatigue, dry mouth, and nausea	Approved for post-AR and post-chemo mCRPC; requires sufficient PSMA expression
PSMA-targeted ADC	Antibody-directed delivery of potent cytotoxic agents	PSA response rates ~15–50% in early trials	Myelosuppression, dryness, and fatigue	Potential salvage or combination strategy; promising new approach under study

Abbreviation: AR, androgen receptor; OS, overall survival; PSA, prostate-specific antigen; mCRPC, metastatic castration-resistant prostate cancer; PCa, prostate cancer; PSMA, prostate-specific membrane antigen; ADC, antibody–drug conjugates.

**Table 3:** PSMA ADC clinical outcomes.

PSMA ADC	Response Rate	Adverse Effects	Toxicity Profile
PSMA-MMAE	PSA ≥ 50% response: 14–21% CTC ≥ 50% response: 45–78%	Neutropenia (36%) Fatigue (34%) Decreased electrolytes (29%) Anemia (25%)	MTD: 2.5 mg/kg DLTs: 2.8 mg/kg
ARX517	PSA ≥ 50% response: 33% ctDNA ≥ 50% response: 50%	Dry mouth (41.7%) Fatigue (33.3%) Diarrhea (20.8%)	N/A
MLN2704	PSA ≥ 50% response: 8–10%	Peripheral neuropathy (71%) Nausea (61%) Fatigue (60%)	N/A
MEDI3726	PSA ≥ 50% response: 3–12%	TRAE (90.9%) Grade 3/4 AEs (45.5%) Elevations in liver enzymes (30.3–36.4%) Fatigue (30.3%)	DLTs: 0.3 mg/kg

Abbreviation: PSMA, prostate-specific membrane antigen; ADC, antibody–drug conjugates; MMAE, monomethyl auristatin E; PSA, prostate-specific antigen; CTC, circulating tumor cell; MTD, maximum tolerated dose; DLT, dose-limiting toxicities; ctDNA, circulating tumor DNA.

## Data Availability

Data available on request/reasonable request.

## References

[1] Rebello R.J., Oing C., Knudsen K.E., Loeb S., Johnson D.C., Reiter R.E., Gillessen S., Van der Kwast T., Bristow R.G. (2021). Prostate cancer. Nat. Rev. Dis. Primers.

[2] Siegel R.L., Miller K.D., Jemal A. (2018). Cancer statistics, 2018. CA Cancer J. Clin..

[3] Henríquez I., Roach M., Morgan T.M., Bossi A., Gómez J.A., Abuchaibe O., Couñago F. (2021). Current and Emerging Therapies for Metastatic Castration-Resistant Prostate Cancer (mCRPC). Biomedicines.

[4] Francini E., Gray K.P., Shaw G.K., Evan C.P., Hamid A.A., Perry C.E., Kantoff P.W., Taplin M.E., Sweeney C.J. (2019). Impact of new systemic therapies on overall survival of patients with metastatic castration-resistant prostate cancer in a hospital-based registry. Prostate Cancer Prostatic Dis..

[5] Chang S.S. (2004). Overview of prostate-specific membrane antigen. Rev. Urol..

[6] Perner S., Hofer M.D., Kim R., Shah R.B., Li H., Möller P., Hautmann R.E., Gschwend J.E., Kuefer R., Rubin M.A. (2007). Prostate-specific membrane antigen expression as a predictor of prostate cancer progression. Hum. Pathol..

[7] Drake P.M., Rabuka D. (2017). Recent Developments in ADC Technology: Preclinical Studies Signal Future Clinical Trends. BioDrugs.

[8] Gogia P., Ashraf H., Bhasin S., Xu Y. (2023). Antibody-Drug Conjugates: A Review of Approved Drugs and Their Clinical Level of Evidence. Cancers.

[9] Shih C.H., Lin Y.H., Luo H.L., Sung W.W. (2024). Antibody-drug conjugates targeting HER2 for the treatment of urothelial carcinoma: Potential therapies for HER2-positive urothelial carcinoma. Front. Pharmacol..

[10] Nagayama A., Ellisen L.W., Chabner B., Bardia A. (2017). Antibody-Drug Conjugates for the Treatment of Solid Tumors: Clinical Experience and Latest Developments. Target. Oncol..

[11] Jain N., Smith S.W., Ghone S., Tomczuk B. (2015). Current ADC Linker Chemistry. Pharm. Res..

[12] Waight A.B., Bargsten K., Doronina S., Steinmetz M.O., Sussman D., Prota A.E. (2016). Structural Basis of Microtubule Destabilization by Potent Auristatin Anti-Mitotics. PLoS ONE.

[13] Voigt W., Matsui S., Yin M.B., Burhans W.C., Minderman H., Rustum Y.M. (1998). Topoisomerase-I inhibitor SN-38 can induce DNA damage and chromosomal aberrations independent from DNA synthesis. Anticancer. Res..

[14] Leyton J.V. (2023). The endosomal-lysosomal system in ADC design and cancer therapy. Expert. Opin. Biol. Ther..

[15] Giugliano F., Corti C., Tarantino P., Michelini F., Curigliano G. (2022). Bystander effect of antibody-drug conjugates: Fact or fiction?. Curr. Oncol. Rep..

[16] Miyahira A.K., Pienta K.J., Morris M.J., Bander N.H., Baum R.P., Fendler W.P., Goeckeler W., Gorin M.A., Hennekes H., Pomper M.G. (2018). Meeting report from the Prostate Cancer Foundation PSMA-directed radionuclide scientific working group. Prostate.

[17] Wittrup K.D., Thurber G.M., Schmidt M.M., Rhoden J.J. (2012). Practical theoretic guidance for the design of tumor-targeting agents. Methods Enzymol..

[18] Lee C.M., Tannock I.F. (2010). The distribution of the therapeutic monoclonal antibodies cetuximab and trastuzumab within solid tumors. BMC Cancer.

[19] Troyer J.K., Feng Q., Beckett M.L., Wright G.L. (1995). Biochemical characterization and mapping of the 7E11-C5.3 epitope of the prostate-specific membrane antigen. Urol. Oncol..

[20] Barren R.J., Holmes E.H., Boynton A.L., Misrock S.L., Murphy G.P. (1997). Monoclonal antibody 7E11.C5 staining of viable LNCaP cells. Prostate.

[21] Aloysius H., Hu L. (2015). Targeted prodrug approaches for hormone refractory prostate cancer. Med. Res. Rev..

[22] Tagawa S.T., Thomas C., Sartor A.O., Sun M., Stangl-Kremser J., Bissassar M., Vallabhajosula S., Huicochea Castellanos S., Nauseef J.T., Sternberg C.N. (2024). Prostate-Specific Membrane Antigen-Targeting Alpha Emitter via Antibody Delivery for Metastatic Castration-Resistant Prostate Cancer: A Phase I Dose-Escalation Study of (225)Ac-J591. J. Clin. Oncol..

[23] Holland J.P., Divilov V., Bander N.H., Smith-Jones P.M., Larson S.M., Lewis J.S. (2010). 89Zr-DFO-J591 for immunoPET of prostate-specific membrane antigen expression in vivo. J. Nucl. Med..

[24] Nováková Z., Foss C.A., Copeland B.T., Morath V., Baranová P., Havlínová B., Skerra A., Pomper M.G., Barinka C. (2017). Novel Monoclonal Antibodies Recognizing Human Prostate-Specific Membrane Antigen (PSMA) as Research and Theranostic Tools. Prostate.

[25] Banerjee S.R., Kumar V., Lisok A., Plyku D., Nováková Z., Brummet M., Wharram B., Barinka C., Hobbs R., Pomper M.G. (2019). Evaluation of (111)In-DOTA-5D3, a Surrogate SPECT Imaging Agent for Radioimmunotherapy of Prostate-Specific Membrane Antigen. J. Nucl. Med..

[26] Petrylak D.P., Kantoff P., Vogelzang N.J., Mega A., Fleming M.T., Stephenson J.J., Frank R., Shore N.D., Dreicer R., McClay E.F. (2019). Phase 1 study of PSMA ADC, an antibody-drug conjugate targeting prostate-specific membrane antigen, in chemotherapy-refractory prostate cancer. Prostate.

[27] Petrylak D.P., Vogelzang N.J., Chatta K., Fleming M.T., Smith D.C., Appleman L.J., Hussain A., Modiano M., Singh P., Tagawa S.T. (2020). PSMA ADC monotherapy in patients with progressive metastatic castration-resistant prostate cancer following abiraterone and/or enzalutamide: Efficacy and safety in open-label single-arm phase 2 study. Prostate.

[28] Skidmore L.K., Mills D., Kim J.Y., Knudsen N.A., Nelson J.D., Pal M., Wang J., Gc K., Gray M.J., Barkho W. (2024). Preclinical Characterization of ARX517, a Site-Specific Stable PSMA-Targeted Antibody-Drug Conjugate for the Treatment of Metastatic Castration-Resistant Prostate Cancer. Mol. Cancer Ther..

[29] Shen J., Pachynski R., Nordquist L.T., Adra N., Bilen M.A., Aggarwal R., Reichert Z., Schweizer M., Iravani A., Aung S. (2023). 1804P APEX-01: First-in-human phase I/II study of ARX517 an anti- prostate-specific membrane antigen (PSMA) antibody-drug conjugate (ADC) in patients (pts) with metastatic castration-resistant prostate cancer (mCRPC). Ann. Oncol..

[30] Doehn C., Jocham D. (2002). Technology evaluation: MLN-591, Cornell University/BZL Biologics/ImmunoGen/Millennium. Curr. Opin. Mol. Ther..

[31] Milowsky M.I., Galsky M.D., Morris M.J., Crona D.J., George D.J., Dreicer R., Tse K., Petruck J., Webb I.J., Bander N.H. (2016). Phase 1/2 multiple ascending dose trial of the prostate-specific membrane antigen-targeted antibody drug conjugate MLN2704 in metastatic castration-resistant prostate cancer. Urol. Oncol..

[32] Cho S., Zammarchi F., Williams D.G., Havenith C.E.G., Monks N.R., Tyrer P., D’Hooge F., Fleming R., Vashisht K., Dimasi N. (2018). Antitumor Activity of MEDI3726 (ADCT-401), a Pyrrolobenzodiazepine Antibody-Drug Conjugate Targeting PSMA, in Preclinical Models of Prostate Cancer. Mol. Cancer Ther..

[33] Adair J.R., Howard P.W., Hartley J.A., Williams D.G., Chester K.A. (2012). Antibody-drug conjugates—A perfect synergy. Expert Opin. Biol. Ther..

[34] de Bono J.S., Fleming M.T., Wang J.S., Cathomas R., Miralles M.S., Bothos J., Hinrichs M.J., Zhang Q., He P., Williams M. (2021). Phase I Study of MEDI3726: A Prostate-Specific Membrane Antigen-Targeted Antibody-Drug Conjugate, in Patients with mCRPC after Failure of Abiraterone or Enzalutamide. Clin. Cancer Res..

[35] de Bono J.S., Scher H.I., Montgomery R.B., Parker C., Miller M.C., Tissing H., Doyle G.V., Terstappen L.W., Pienta K.J., Raghavan D. (2008). Circulating tumor cells predict survival benefit from treatment in metastatic castration-resistant prostate cancer. Clin. Cancer Res..

[36] Zhang D., Yu S.F., Khojasteh S.C., Ma Y., Pillow T.H., Sadowsky J.D., Su D., Kozak K.R., Xu K., Polson A.G. (2018). Intratumoral Payload Concentration Correlates with the Activity of Antibody-Drug Conjugates. Mol. Cancer Ther..

[37] Su D., Zhang D. (2021). Linker Design Impacts Antibody-Drug Conjugate Pharmacokinetics and Efficacy via Modulating the Stability and Payload Release Efficiency. Front. Pharmacol..

[38] Johann F., Wöll S., Gieseler H. (2024). “Negative” Impact: The Role of Payload Charge in the Physicochemical Stability of Auristatin Antibody–Drug Conjugates. J. Pharm. Sci..

[39] Wang Z., Li H., Gou L., Li W., Wang Y. (2023). Antibody-drug conjugates: Recent advances in payloads. Acta Pharm. Sin. B.

[40] de Bono J.S., Logothetis C.J., Molina A., Fizazi K., North S., Chu L., Chi K.N., Jones R.J., Goodman O.B., Saad F. (2011). Abiraterone and increased survival in metastatic prostate cancer. N. Engl. J. Med..

[41] Berthold D.R., Pond G.R., Soban F., de Wit R., Eisenberger M., Tannock I.F. (2008). Docetaxel plus prednisone or mitoxantrone plus prednisone for advanced prostate cancer: Updated survival in the TAX 327 study. J. Clin. Oncol..

[42] Sartor O., de Bono J., Chi K.N., Fizazi K., Herrmann K., Rahbar K., Tagawa S.T., Nordquist L.T., Vaishampayan N., El-Haddad G. (2021). Lutetium-177-PSMA-617 for Metastatic Castration-Resistant Prostate Cancer. N. Engl. J. Med..

[43] Alati S., Singh R., Pomper M.G., Rowe S.P., Banerjee S.R. (2023). Preclinical Development in Radiopharmaceutical Therapy for Prostate Cancer. Semin. Nucl. Med..

[44] Hennrich U., Eder M. (2022). [^177^Lu]Lu-PSMA-617 (Pluvicto^TM^): The First FDA-Approved Radiotherapeutical for Treatment of Prostate Cancer. Pharmaceuticals.

[45] Hofman M.S., Violet J., Hicks R.J., Ferdinandus J., Thang S.P., Akhurst T., Iravani A., Kong G., Ravi Kumar A., Murphy D.G. (2018). [^177^Lu]-PSMA-617 radionuclide treatment in patients with metastatic castration-resistant prostate cancer (LuPSMA trial): A single-centre, single-arm, phase 2 study. Lancet Oncol..

[46] Heynickx N., Herrmann K., Vermeulen K., Baatout S., Aerts A. (2021). The salivary glands as a dose limiting organ of PSMA- targeted radionuclide therapy: A review of the lessons learnt so far. Nucl. Med. Biol..

[47] Chi K.N., Yip S.M., Bauman G., Probst S., Emmenegger U., Kollmannsberger C.K., Martineau P., Niazi T., Pouliot F., Rendon R. (2024). ^177^Lu-PSMA-617 in Metastatic Castration-Resistant Prostate Cancer: A Review of the Evidence and Implications for Canadian Clinical Practice. Curr. Oncol..

[48] Nambiar D.K., Mishra D., Singh R.P. (2023). Targeting DNA repair for cancer treatment: Lessons from PARP inhibitor trials. Oncol. Res..

[49] Salifu I., Singh N., Berraondo M., Remon J., Salifu S., Severson E., Quintana A., Peiró S., Ramkissoon S., Vidal L. (2023). Antibody-drug conjugates, immune-checkpoint inhibitors, and their combination in advanced non-small cell lung cancer. Cancer Treat. Res. Commun..

[50] Dale D.C. (2009). Advances in the treatment of neutropenia. Curr. Opin. Support. Palliat. Care.

[51] McCombs J.R., Owen S.C. (2015). Antibody drug conjugates: Design and selection of linker, payload and conjugation chemistry. AAPS J..

[52] Ferraro D.A., Rüschoff J.H., Muehlematter U.J., Kranzbühler B., Müller J., Messerli M., Husmann L., Hermanns T., Eberli D., Rupp N.J. (2020). Immunohistochemical PSMA expression patterns of primary prostate cancer tissue are associated with the detection rate of biochemical recurrence with (68)Ga-PSMA-11-PET. Theranostics.

[53] Sommer U., Siciliano T., Ebersbach C., Beier A.K., Stope M.B., Jöhrens K., Baretton G.B., Borkowetz A., Thomas C., Erb H.H.H. (2022). Impact of Androgen Receptor Activity on Prostate-Specific Membrane Antigen Expression in Prostate Cancer Cells. Int. J. Mol. Sci..

